# Attentional factorization machine with review-based user–item interaction for recommendation

**DOI:** 10.1038/s41598-023-40633-4

**Published:** 2023-08-18

**Authors:** Zheng Li, Di Jin, Ke Yuan

**Affiliations:** 1 College of Computer and Information Engineering, Henan University, Kaifeng, 475004 Henan China; 2 Henan Engineering Laboratory of Spatial Information Processing, Henan University, Kaifeng, 475004 Henan China; 3 Henan Key Laboratory of Big Data Analysis and Processing, Henan University, Kaifeng, 475004 Henan China

**Keywords:** Information technology, Scientific data

## Abstract

In recommender systems, user reviews on items contain rich semantic information, which can express users’ preferences and item features. However, existing review-based recommendation methods either use the static word vector model or cannot effectively extract long sequence features in reviews, resulting in the limited ability of user feature expression. Furthermore, the impact of different or useless feature interactions between users and items on recommendation performance is ignored. Therefore, we propose an attentional factorization machine with review-based user–item interaction for recommendation (AFMRUI), which first leverages RoBERTa to obtain the embedding feature of each user/item review, and combines bidirectional gated recurrent units with attention network to highlight more useful information in both user and item reviews. Then we adopt AFM to learn user–item feature interactions to distinguish the importance of different user–item feature interactions and further to obtain more accurate rating prediction, so as to promote recommendation. Finally, we conducted performance evaluation on five real-world datasets. Experimental results on five datasets demonstrated that the proposed AFMRUI outperformed the state-of-the-art review-based methods regarding two commonly used evaluation metrics.

## Introduction

With the rapid development of Internet industry and big data technology, recommender systems are playing an increasingly important role in social networks^1^, academic education^2^, e-commerce^3^, and so on. Nowadays, recommender systems have become an indispensable part of daily life, such as online shopping^4^, next point-of-interest recommendation^5^, music recommendation^6^, and video push^7^. According to users’ historical behavioral data, recommender systems can predict users’ ratings of items and perform personalized recommendation, so as to help users quickly discover items they are interested in and improve users’ satisfaction. Therefore, in order to provide better personalized recommendation services, how to accurately predict users’ ratings on items to boost recommendation becomes a challenge problem.

To solve the above issue, researchers have proposed a variety of item rating prediction methods, among which rating prediction method^8^ based on collaborative filtering (CF) is one of the most widely used methods. Most CF methods are based on matrix factorization^9,10^, learning latent features of users and items from matrix models for recommendation. Considering users ratings for items reflect their interaction behaviors and explicit features, Zhang et al.^11^ obtained users and items features from user–item rating information based on deep matrix factorization. However, with the rapid growth of the number of users and items, there are more and more problems such as sparsity of the rating data. Unfortunately, the information extracted from rating data is limited, consequently restricting the recommendation performance.

Compared with rating data, review information contains rich semantics, which can not only reflect users’ satisfaction with item quality and function, but also indirectly express users’ preferences and item features^12^. Thus, review-based item rating prediction has attracted extensive attention from researchers, such as ConvMF^13^, DeepCoNN^14^, D-Attn^15^, NARRE^16^, and DAML^17^, etc. These methods can alleviate the sparsity problem caused by rating data through review information, and thus obtain relatively accurate prediction ratings for recommendation. However, there are two major limitations as follows:

1. The expression ability of user/item features is insufficient. In above research, D-Attn^15^, DAML^17^, etc., leverage word vectors statically encoded such as word2vec or Glove, resulting in sparse feature representation, insufficient semantics and polysemy, which affect the ability of model to extract user and item features. Moreover, models such as ConvMF^13^, DeepCoNN^14^, and NARRE^16^ use convolutional neural networks (CNN) to extract users and items features from reviews, which cannot effectively extract long sequence text features in reviews, and thus cannot accurately express user or item features, limiting the model performance.
2. The influence of feature interactions between users and items on the recommendation performance is ignored. For example, models, such as DeepCoNN^14^, D-Attn^15^, NARRE^16^, DAML^17^, etc., obtain prediction ratings by dot product or factorization machine after splicing of users and items features. Such feature interaction modelling methods ignore different effects of different feature interactions on recommendation results. Furthermore, useless feature interactions will introduce noise, thus reducing the recommendation performance.

To address the above issues, this paper proposed an attentional factorization machine with review-based user–item interaction for recommendation. Specifically, in order to better capture review-based user features and item features, we first obtain the embedding feature of each review through the pre-trained model RoBERTa, which alleviates the problem that static word vectors cannot adapt to polysemy; then we combine bidirectional gate recurrent unit (BiGRU) and attention network to highlight important information in reviews, and obtain user reviews embedding and item reviews embedding; furthermore, the obtained reviews embedding of user and item are concatenated together and input to attentional factorization machine (AFM) to perform more accurately rating prediction, so as to make recommendation. The main contributions of this paper can be summarized as follows:

- We build an enhanced framework for user/item feature representation, which leverages RoBERTa to obtain the embedding feature of each user/item review to alleviate the problem of polysemy, and uses BiGRU and attention network to measure the contribution of embedding feature of each review, so as to obtain better expression ability of user/item features;
- We use AFM to learn user–item feature interactions and to distinguish the importance of different feature interactions, which can further alleviate the effect of noise that may be introduced by useless feature interactions;
- We conduct comprehensive experiments on five real-world datasets, which demonstrate that our proposed AFMRUI model outperforms the state-of-the-art models.

The remainder of this paper is organized as follows. In “Related work”, we provide an overview of related work. Section “The proposed approach” elaborates our proposed AFMRUI model. Next, we evaluate the effectiveness of our model and analyze the experimental results in “Experiments”. Finally, “Conclusions” presents the conclusions and sketches directions for future work.

## Related work

### Embedding representation methods

In review-based recommendation tasks, word embedding representation methods are usually used to express user or item review embedding features. Models, such as ConvMF^13^, DeepCoNN^14^, D-Attn^15^, NARRE^16^, and DAML^17^, etc., use Glove^18^ and Word2Vec^19^ belonging to static word vector models. However, the obtained user/item review embedding features cannot change with the contextual semantics, and the problem of polysemy will be produced. As a result, dynamic word vectors are used to solve the problem. For example, Google proposed Bidirectional Encoder Representation from Transformers (BERT)^20^, a dynamic word vector pre-trained model, to achieve excellent results in 11 natural language processing tasks. In recent research, SIFN^21^ and U-BERT^22^ use BERT to obtain the review embedding representation, which have a large performance improvement in rating prediction compared with methods using static word vector models.

Based on BERT, an improved model RoBERTa^23^ was introduced, which not only inherits the advantages of BERT, but also simplifies the next sentence prediction task in BERT. RoBERTa is retrained using new hyperparameters and a large new dataset, which allows the model to be more fully trained and has a significant improvement in performance. To this end, we adopt RoBERTa in our model to mitigate the problem of polysemy in user/item reviews by encoding the obtained word-level embedding representation of each review.

### Review-based recommendation methods

With the increase of interactive information generated by users in various fields, various interactive information related to users and items, e.g., reviews, is introduced into the recommender system to improve the performance. Next, we will outline two review-based recommendation methods.

#### Review-based topic modeling recommendation methods

Topic modeling approaches were the first to apply reviews to recommender systems, mainly obtaining the latent topic distribution in reviews through latent dirichlet allocation (LDA) or non-negative matrix factorization, and demonstrated the usefulness of reviews. For example, Xu et al.^24^ proposed a topic model-based CF model, which mainly obtained review-based features through an LDA-based extended model. Huang et al.^25^ similarly obtained potential features of users in Yelp restaurant review dataset by LDA algorithm, which can help restaurant operators understand customer preferences. Since the topic model based on LDA cannot preserve the word order information, the context information in the reviews is ignored.

Aiming at the problems of LDA algorithm, Bao et al.^26^ proposed a TopicMF model, which used the latent factors of users and items obtained by matrix factorization to correlate, so as to improve the accuracy of rating prediction. Ganu et al.^27^ learned preference features of each user from reviews information, and used a CF method based on latent factor model (LFM) for rating prediction. However, LFM model can only learn those linear and low-level features, which is not conducive to interactive learning among features from fusion layers.

The methods mentioned above use the bag-of-words-based topic model for review processing, which cannot preserve the word order information well, so that the local context information contained in reviews will be ignored, and only shallow semantic information can be extracted. However, the rich semantic information in user/item reviews cannot be accurately captured. While in our research, we use RoBERTa and BiGRU to model user reviews and item reviews, so as to effectively obtain user and item review embedding features with rich semantics.

#### Review-based deep learning recommendation methods

In recent years, CNN has been widely used in the task of review-based recommendation. For example, Kim et al.^13^ first introduced CNN into recommender system and proposed ConvMF model. However, ConvMF model only uses item reviews and user ratings during training, ignoring user reviews information. For this problem, Zheng et al.^12^ introduced a deep parallel network framework DeepCoNN, which alleviated the problems in ConvMF by using two parallel CNN networks to model user review documents and item review documents respectively. Considering that different words have different importance for modeling users and items, Seo et al.^15^ introduced CNN with dual local and global attention to learn reviews embedding of each user and each item, so as to perform rating prediction. Chen et al.^16^ introduced a neural attentional regression model with review-level explanations, which used a review-level attention mechanism to assign different weights to each review, making the recommendation interpretable. The above methods use CNN to encode reviews, but CNN-based methods fail to effectively extract features from reviews with different lengths.

To address the above problem, Tay et al.^28^ learned feature representations of users and items by using pointers at the word-level and review-level based on review information, to obtain important information in reviews to improve the prediction results. Chen et al.^29^ modeled dynamic preferences of users as well as item attributes through gated recurrent unit (GRU) and sentence-level CNN, and improved the interpretability of the proposed model.

According to the above analysis, review-based deep learning recommendation methods have superior performance compared with topic-based modeling recommendation methods. So in our model, we leverage BiGRU and incorporate attention network to measure the importance of each review, so as to improve user/item feature representations.

### Feature interaction methods

For the feature interactions between users and items, some research uses traditional feature interaction methods, such as dot product^30^, fully connected^31^, factorization machines (FM)^32^, etc. FM are supervised learning methods that augment linear regression models by incorporating feature interactions. For example, multi-pointer co-attention networks^28^ shows that FM obtain better results than other interaction models for its good interaction ability. However, traditional methods model all feature interactions and fail to distinguish the importance of different feature interactions. Therefore, Zhang et al.^33^ proposed a combination model of FM and deep neural network based on factorization machine neural network model, which generated higher-order feature combinations, and strengthened the learning ability of models to features.

However, for different samples, the weights of different feature interactions should also be different. In other words, for those unimportant feature interactions, it should reduce their weights. While for those high-importance feature interactions, it should increase their weights. To this end, Xiao et al.^34^ improved FM by recognizing the importance of different feature interactions, and introduced an AFM, which can learn the importance of feature interactions through attention mechanism, so as to alleviate the problem of reduced feature representations performance caused by those useless feature interactions.

Inspired by reference^34^, our AFMRUI model adopt AFM to learn the feature interactions of users and items, and obtain better feature representations by distinguishing the importance of different feature interactions, and alleviate the effect of noise that may be introduced by useless feature interactions.

## The proposed approach

In this section, we first present the problem definition of our recommendation task and list key notations used in our work in Table 1, and then elaborate the model framework of AFMRUI.

### Problem definition

Assume that dataset *D* contains *M* users and *N* items as well as plentiful reviews and the corresponding ratings. Each sample in dataset *D* is defined as userID-itemID-review-rating quadruplet (*u*, *i*, *r*, *y*(*x*)), meaning that user *u* makes a review *r* and gives the corresponding rating *y*(*x*) on item *i*. For all samples in dataset *D*, we can obtain the review set of each user and the review set of each item by retrieving userID and itemID. In this work, we focus on predicting a user’s rating on an item based on the obtained corresponding review sets of user and item. We define the review-based recommendation task as follows:

**Table 1:** Key notations used in this paper.

Notation	Interpretation
*M, N*	Total number of users, total number of items in a dataset
*u, i*	A specific user, an item
*n, m*	User’s maximum number of reviews, item’s maximum number of reviews
*D*$_u$, *D*$_i$	Review set of user *u*, review set of item *i*
*RD*$_u$	Reviews list of user *u* after preprocessing
*RD*$_i$	Reviews list of item *i* after preprocessing
${\mathrm{{r}}_{{u_n}}}$	The word-level embedding representation of user review ${d_{{u_n}}}$ from *RD*$_u$
${\mathrm{{r}}_{{i_m}}}$	The word-level embedding representation of item review ${d_{{i_m}}}$ from *RD*$_i$
${{\textbf{O}}_u}$	The embedding feature list of user reviews obtained by RoBERTa
${{\textbf{O}}_i}$	The embedding feature list of item reviews obtained by RoBERTa
${{\textbf{H}}_u}$	The whole hidden feature of user review extracted from sequence coding layer
${{\textbf{H}}_i}$	The whole hidden feature of item review extracted from sequence coding layer
${\alpha _u}$	Attention weights vector corresponding to the whole hidden feature ${{\textbf{H}}_u}$
${\alpha _i}$	Attention weights vector corresponding to the whole hidden feature ${{\textbf{H}}_i}$
${{\text {R}}_u}$	Review embedding of user *u* obtained by attention layer
${{\text {R}}_i}$	Review embedding of item *i* obtained by attention layer
x	The joint vector of user review embedding and item review embedding
${\hat{y}}(x)$	The predicted user *u*’s rating of item *i*

Definition (review-based recommendation task). Given a review set *D*$_u$ of user *u* and a review set *D*$_i$ of an item *i*, the task of review-based recommendation is to predict user *u*’s rating $\hat{y}(x)$ on the item *i* and then makes recommendation.

### AFMRUI framework

**Figure 1:**
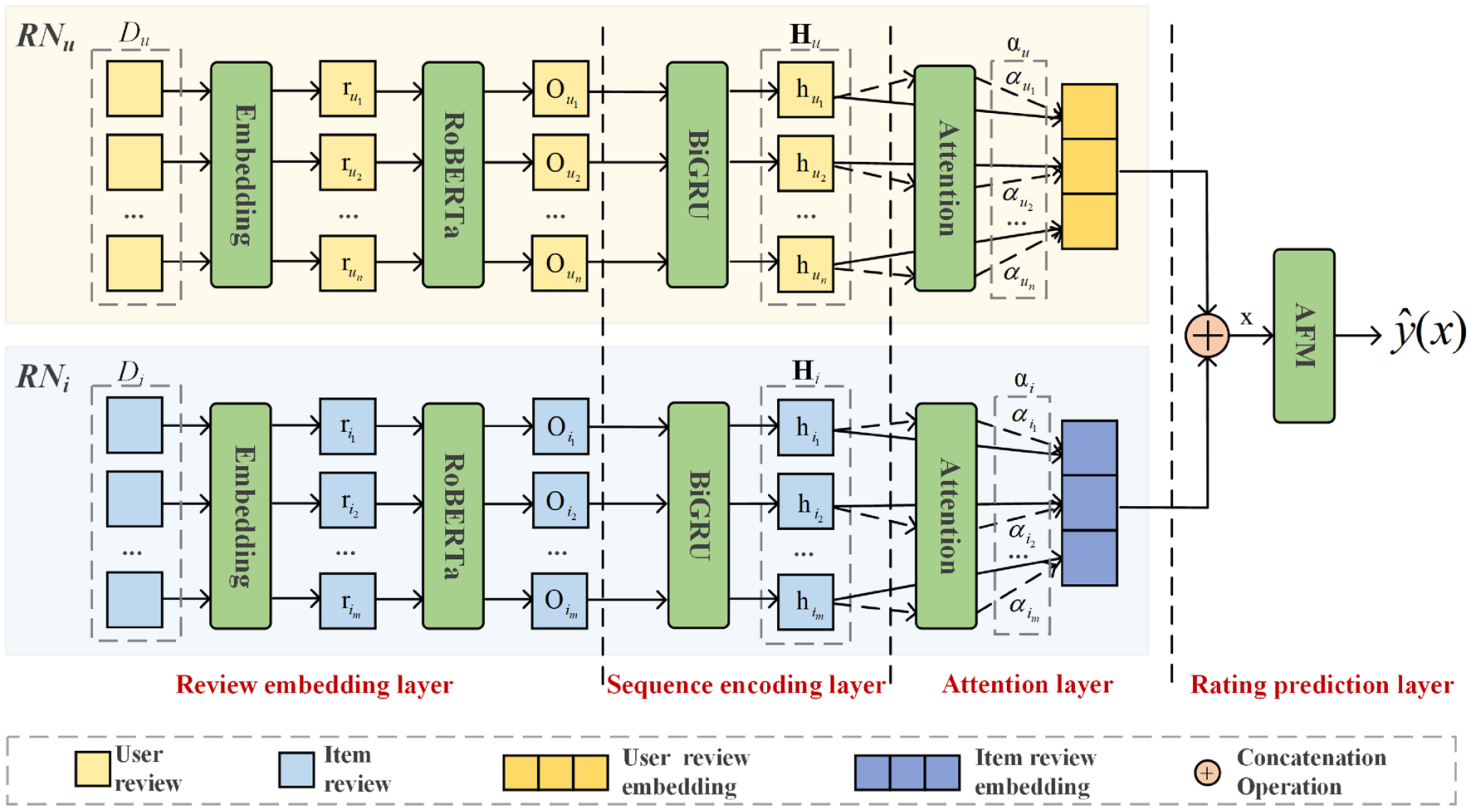
Illustration of AFMRUI model.

The architecture of the proposed AFMRUI model is shown in Fig. 1. The AFMRUI model is composed of two parallel networks with similar structures, namely, user review network *RN*$_u$ and item review network *RN*$_i$. Review set *D*$_u$ of a user *u* and review set *D*$_i$ of an item *i* are given to *RN*$_u$ and *RN*$_i$ respectively as inputs, and the corresponding rating predicted on item *i* is produced as the output, so as to make recommendation. It can be seen from Fig. 1, AFMRUI model consists of four layers. Each layer is outlined as follows:

1. Review embedding layer, which is mainly used to obtain the embedding feature of each review from the sets *D*$_\textit{u}$ and *D*$_i$ by RoBERTa;
2. Sequence encoding layer, which mainly leverages BiGRU to encode embedding feature of each review produced by review embedding layer, and fully mines the internal dependencies among review embedding features, so as to obtain the corresponding hidden features;
3. Attention layer, which is utilized to obtain reviews embedding of a user or an item by adaptively measuring the weight of hidden feature of each review, so that the model can focus on more useful reviews and improve the feature expression ability of users and items;
4. Rating prediction layer, which first concatenates the reviews embedding of user *u* and item *i* obtained from attention layer, and further leverages AFM to learn user–item feature interactions to predict user *u*’s rating on item *i*, and then makes recommendation.
  Since *RN*$_u$ and *RN*$_i$ only differ in their inputs, so next we take *RN*$_u$ network as an example to illustrate the process in detail. Note that the process described in the following subsections “Review embedding layer”, “Sequence encoding layer”, and “Attention layer” is also applied to *RN*$_i$ network.

#### Review embedding layer

Review embedding layer is used to obtain embedding feature of each review from user review set *D*$_u$ by RoBERTa. According to the requirements of RoBERTa, the original reviews from *D*$_u$ need to be preprocessed to achieve the corresponding review embedding features.

Specifically, we first remove special characters, such as mathematical symbols, punctuation marks, in each review from *D*$_u$, and set the obtained reviews to a unified maximum length. Then, we combine each review processed into a list to get the corresponding user review list *RL*$_u$. Furthermore, we set the obtained review list of each user in the dataset to a fixed length *n*, where *n* represents users’ maximum number of reviews input to RoBERTa. If the length of *RL*$_u$ exceeds *n*, the truncation operation is performed to get the first *n* reviews in *RL*$_u$. Otherwise, we use zero vectors for filling operation after RoBERTa mapping to get the specified length *n*. Afterwards, we insert special characters <s> and </s> at the beginning and end of each review respectively after fixed length processing to obtain review list *RD*$_u$ of user *u*, denoted as $\{ {d_{{u_1}}},{d_{{u_2}}},\ldots ,{d_{{u_n}}}\}$.

**Figure 2:**
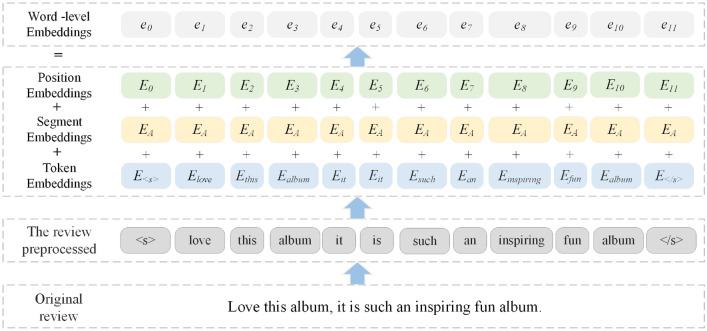
Illustration how to obtain the word-level embedding representation of a review.

Subsequently, each review in the list *RD*$_u$ needs to be expressed in the form of word-level embedding representation, which is composed of token embeddings, segment embeddings and position embeddings. Take the review “Love this album. It is such an inspiring fun album”. by user A2B2J5VS139VLM on item B004L49K20 in Digital Music dataset as an example. Figure 2 shows how to obtain the word-level embedding representation of the review.

As shown in Fig. 2, the original review is preprocessed as the input of word-level embedding representation. Then we extract token embeddings, segment embeddings and position embeddings from the preprocessed review respectively, and then add them to get the word-level embedding representation of the review. For the *f*-th token in the preprocessed user review ${d_{{u_i}}}$, its word-level embedding representation is denoted as:

1 $$\begin{aligned} e_{f}=E_{\textit{token}(f)}+E_{\textit{seg}(f)}+E_{\textit{pos}(f)} \end{aligned}$$

where *E*$_{token(f)}$ is the token embedding corresponding to the *f*-th token in ${d_{{u_i}}}$, which is obtained by mapping the token as a 768-dimensional embedding; *E*$_{seg(f)}$ represents the segment embedding corresponding to the *f*-th token in ${d_{{u_i}}}$. Since each preprocessed review can be considered as a sentence, so the segment embedding of each word in ${d_{{u_i}}}$ is the same. As shown in the “segment embeddings” in Fig. 2, the segment embedding of each token from the review in the example is *E*$_A$; *E*$_{pos(f)}$ is the position embedding, which represents the result of encoding the position of each word in ${d_{{u_i}}}$.

Based on the above processing, we can obtain ${\mathrm{{r}}_{{u_i}}}$, the word-level embedding representation of ${d_{{u_i}}}$ from the list *RD*$_u$, which is represented as:

2 $$\begin{aligned} {\mathrm{{r}}_{{u_i}}} = [{e_0},{e_1},\ldots ,{e_j}] \end{aligned}$$

By doing the same operation for each preprocessed review from *RD*$_u$, we obtain the corresponding word-level embedding representation of each review, represented as $\{ {\mathrm{{r}}_{{u_1}}},{\mathrm{{r}}_{{u_2}}},\ldots ,{\mathrm{{r}}_{{u_n}}}\}$, where *n* represents the specified maximum number of user reviews.

Considering the multi-head attention mechanism in RoBERTa can effectively capture the semantic information among tokens in a review, which can mitigate the problem of polysemy in user/item reviews. Therefore, we leverage RoBERTa to semantically encode the obtained word-level embedding representation of each review. Specifically, given the word-level review embedding representation ${\mathrm{{r}}_{{u_i}}}$ as the input of RoBERTa, we can obtain the corresponding review embedding feature ${\mathrm{{O}}_{{u_i}}}$, denoted as:

3 $$\begin{aligned} {\mathrm{{O}}_{{u_i}}} = \mathrm{{RoBERTa}}({\mathrm{{r}}_{{u_i}}}),i = 1,2,\ldots ,n \end{aligned}$$

where ${\mathrm{{O}}_{{u_i}}}$ is a fixed *c*-dimensional semantic feature.

Then the embedding features of reviews from *RD*$_u$ output by RoBERTa can be represented by a review embedding feature list ${{\textbf{O}}_u} \in {\mathbb {R}^{n \times c}}$, denoted as ${{\{ }}{{\text {O}}_{{u_1}}},{{\text {O}}_{{u_2}}},\ldots ,{{\text {O}}_{{u_n}}}\}$.

#### Sequence encoding layer

Sequence encoding layer is used to obtain the corresponding hidden features of each review. In order to capture the relationships among review embedding features of user *u*, we use BiGRU, which has proven to be successful in practical applications^35^$$^,$$^36^, to encode embedding feature of each review from list $${{\textbf{O}}_u}$$. In this way, embedding feature of each review can be modeled from forward and backward directions, and fully mines the internal dependencies among review embedding features, so as to obtain the corresponding hidden features.

Specifically, we take the list ${{\{ }}{{\text {O}}_{{u_1}}},{{\text {O}}_{{u_2}}},\ldots ,{{\text {O}}_{{u_n}}}\}$ as the input of BiGRU to obtain the corresponding forward hidden feature and backward hidden feature, represented as:

4 $$\begin{aligned} \overrightarrow{{\mathrm{{h}}_{{u_i}}}}= & {} \overrightarrow{GRU} \left( {{\mathrm{{O}}_{{u_i}}},\overrightarrow{{\mathrm{{h}}_{{u_{i - 1}}}}} } \right) \end{aligned}$$

5 $$\begin{aligned} \overleftarrow{{\mathrm{{h}}_{{u_i}}}}= & {} \overleftarrow{GRU} \left( {{\mathrm{{O}}_{{u_i}}},\overleftarrow{{\mathrm{{h}}_{{u_{i + 1}}}}} } \right) \end{aligned}$$

where $\overrightarrow{{\mathrm{{h}}_{{u_i}}}}$ represents the forward hidden feature corresponding to ${\mathrm{{O}}_{{u_i}}}$, $\overrightarrow{GRU}$ represents forward processing from ${\mathrm{{O}}_{{u_1}}}$ to ${\mathrm{{O}}_{{u_n}}}$, $\overrightarrow{{\mathrm{{h}}_{{u_{i - 1}}}}}$ represents the forward hidden feature corresponding to ${\mathrm{{O}}_{{u_{i - 1}}}}$; correspondingly, $\overleftarrow{{\mathrm{{h}}_{{u_i}}}}$ represents the backward hidden feature corresponding to ${\mathrm{{O}}_{{u_i}}}$, $\overleftarrow{GRU}$ represents backward processing from ${\mathrm{{O}}_{{u_n}}}$ to ${\mathrm{{O}}_{{u_1}}}$, $\overleftarrow{{\mathrm{{h}}_{{u_{i + 1}}}}}$ represents the backward hidden feature corresponding to ${\mathrm{{O}}_{{u_{i + 1}}}}$.

Then we concatenate $\overrightarrow{{\mathrm{{h}}_{{u_i}}}}$ with $\overleftarrow{{\mathrm{{h}}_{{u_i}}}}$ of each review to obtain the corresponding hidden feature ${{\text {h}}_{{u_i}}} \in {\mathbb {R}^{2l}}$, where *l* represents the hidden dimension of each GRU. ${{\text {h}}_{{u_i}}}$ is denoted as:

6 $$\begin{aligned} {{\text {h}}_{{u_i}}} = [\overrightarrow{{{\text {h}}_{{u_i}}}} ,\overleftarrow{{{\text {h}}_{{u_i}}}} ] \end{aligned}$$

Similarly, we can obtain the whole hidden feature ${{\textbf{H}}_u} \in {\mathbb {R}^{n \times 2l}}$ corresponding to list ${{\textbf{O}}_u}$ through the sequence coding layer, denoted as:

7 $$\begin{aligned} {{\textbf{H}}_u} = ({{\text {h}}_{{u_1}}},{{\text {h}}_{{u_2}}},\ldots ,{{\text {h}}_{{u_n}}}) \end{aligned}$$

#### Attention layer

Considering reviews made by users on different items reflect different user preferences, we introduce attention mechanism^37^$$^,$$^38^ to adaptively measure weights to review hidden features, and aggregate those more useful informative reviews to form a user review embedding.

Specifically, the attention network takes the whole hidden feature ${{\textbf{H}}_u}$ as input, and yields a corresponding vector of attention weights, ${\alpha _u}\in {\mathbb {R}^{1\times n}}$, represented as:

8 $$\begin{aligned} {\alpha _u} = {\text {soft}}\max ({{\text {w}}_1} \times \tanh ({{\textbf{W}}_1} \times {\textbf{H}}_u^{\text {T}})) \end{aligned}$$

where ${{\text {w}}_1} \in {\mathbb {R}^{1 \times {t_1}}}$ represents a vector of parameters, ${{\textbf{W}}_1} \in {\mathbb {R}^{{t_1} \times 2l}}$ is weight matrix, ${t_1}$ represents the hidden unit number in the attention network. ${\text {soft}}\max ( \cdot )$ is used to normalize the attention weights vector. Each dimension in ${\alpha _u}$ denotes the degree of user preference reflected by each review.

Then, we compute the weighted sums by multiplying attention weights vector ${\alpha _u}$ and whole hidden feature ${{\textbf{H}}_u}$, to obtain user review vector ${{\text {d}}_u} \in {\mathbb {R}^{1 \times 2l}}$, denoted as:

9 $$\begin{aligned} {{\text {d}}_u} = {\alpha _u}{{\textbf{H}}_u} \end{aligned}$$

Next, ${{\text {d}}_u}$ is used as the input of the fully connected layer to obtain user *u*’s review embedding ${{\text {R}}_u} \in {\mathbb {R}^k}$, where *k* represents the latent dimension. ${{\text {R}}_u}$ is represented as:

10 $$\begin{aligned} {{\text {R}}_u} = {{\textbf{W}}_2} \times {{\text {d}}_u} + {{\text {b}}_1} \end{aligned}$$

where ${{\textbf{W}}_2} \in {\mathbb {R}^{k \times 2l}}$ is the weight matrix of the fully connected layer, and ${{\text {b}}_1} \in {\mathbb {R}^k}$ is a bias term.

Similarly, for *RN*$_i$ network, we can get item *i*’s review embedding ${{\text {R}}_i}$ from the corresponding item review set *D*$_i$.

#### Rating prediction layer

In rating prediction layer, our goal is to predict user *u*’s rating $\hat{y}(x)$ of item *i* based on user review embedding ${{\text {R}}_u}$ and item review embedding ${{\text {R}}_i}$. In fact, the predicted user’s rating of an item is actually a kind of user–item feature interactions. However, most existing approaches, such as dot product, cannot effectively learn user–item feature interactions and fail to distinguish the importance of different feature interactions. While AFM can obtain more accurate rating prediction by distinguishing the importance of different feature interactions, and alleviate the influence of noise that may be introduced by those useless feature interactions. Therefore, we adopt AFM to learn user–item feature interactions and obtain $\hat{y}(x)$.

Specifically, we concatenate ${{\text {R}}_u} \in {\mathbb {R}^k}$ with ${{\text {R}}_i} \in {\mathbb {R}^k}$ into a joint vector ${\text {x}} = ({x_1},{x_2},\ldots ,{x_{2k}})$. Given ${\text {x}} \in {\mathbb {R}^{2k}}$ as input of AFM, it outputs the predicted rating $\hat{y}(x)$, and ensures that each user–item feature interaction in the joint vector reflects different importance. $\hat{y}(x)$ is represented as:

11 $$\begin{aligned} \hat{y}(x) = {w_0} + \sum \limits _{i = 1}^{|\mathrm{{x}}|} {{w_i}{x_i}} + {\mathrm{{p}}^\mathrm{{T}}}\sum \limits _{i = 1}^{|\mathrm{{x}}|} {\sum \limits _{j = i + 1}^{|\mathrm{{x}}|} {{\alpha _{ij}}({\mathrm{{v}}_i} \otimes {\mathrm{{v}}_j}){x_i}{x_j}} } + {b_u} + {b_i} \end{aligned}$$

where ${w_0}$ denotes the global bias term, ${w_i}$ is the weight of the primary term, ${\text {|x|}}$ represents the feature number of the joint vector ${\text {x}}$. ${\text {p}} \in {\mathbb {R}^d}$ represents the weights vector for rating prediction layer. ${{\text {v}}_i} \in {\mathbb {R}^d}$ is an embedding vector corresponding to a certain dimension ${x_i}$. Similarly, ${{\text {v}}_j} \in {\mathbb {R}^d}$ is an embedding vector corresponding to a certain dimension ${x_j}$, and *d* is the size of embedding vector. ${b_u}$ represents the user bias term, and ${b_i}$ represents the item bias term. $\otimes$ represents the element-wise product of embedding vectors, ${\alpha _{ij}}$ represents the attention weight, which is calculated by:

12 $$\begin{aligned} {\alpha _{ij}} = \frac{{\exp \left( {\alpha _{ij}^{'}} \right) }}{{\sum \nolimits _{i,j \in |{\text {x|}},j > i} {\exp \left( {\alpha _{ij}^{'}} \right) } }} \end{aligned}$$

where ${\alpha _{ij}^{'}}$ represents the attention score of the feature interaction of ${x_i}$ and ${x_j}\left( {i,j \in \left| {\text {x}} \right| ,j > i} \right)$, which is computed by:

13 $$\begin{aligned} {\alpha _{ij}^{'}} = {{\text {h}}^{\text {T}}}{\text {Re}} {\text {LU}}({\textbf{W}}({{\text {v}}_i} \otimes {{\text {v}}_j}){x_i}{x_j} + {\text {b}}) \end{aligned}$$

where ${\text {h}} \in {\mathbb {R}^t}$ represents the weights vector from the fully connected layer to the softmax output layer, *t* represents the size of hidden layer of the attention network in AFM. ${\textbf{W}} \in {\mathbb {R}^{t \times d}}$, ${\text {b}} \in {\mathbb {R}^t}$ represent the weight matrix, the bias term, respectively.

On the basis of above operations, item recommendation can be performed according to the obtained predicted ratings.

### Model learning

The squared loss function is widely used in the rating prediction task of the recommender system, so we adopt this loss function, defined as:

14 $$\begin{aligned} L = \sum \limits _{z \in S} {{{(\hat{y}(z) - y(z))}^2}} \end{aligned}$$

where *S* represents the training samples, $\hat{y}(z)$ represents the predicted rating of a sample *z*, and *y*(*z*) represents the real rating of sample *z*.

## Experiments

In this section, we conduct experiments to evaluate the effectiveness of our proposed AFMRUI model on five real-world datasets. We first introduce the experimental setup, including datasets and preprocessing, evaluation metrics, baseline methods and experimental configuration. Afterwards, we conduct the performance comparisons and also demonstrate the corresponding ablation studies. Furthermore, we analyze the effects of different parameters on the performance of AFMRUI and discuss the impacts of different embedding representation methods and different feature interaction methods on model performance.

### Experimental setup

#### Datasets and preprocessing

We evaluate the AFMRUI model on five real-world datasets with different scales and industries. Among them, four Amazon datasets, including Digital Music, Baby, Office Products and Beauty, contain real Amazon reviews from May 1996 to July 2014, and Yelp dataset for the Yelp Challenge. Each sample in each dataset includes userID, itemID, review, ratings, etc. Moreover, users in each dataset have posted at least five reviews on the corresponding items. Table 2 shows the statistics of five datasets.

**Table 2:** Statistics of five datasets.

Datasets	Users	Items	Samples
Digital music	5541	3568	64,706
Baby	19,445	7050	160,792
Office products	4905	2420	53,258
Beauty	22,363	12,101	198,502
Yelp	1,144,046	174,013	5,000,000
Average	239,260	39,830	1,095,452

To ensure the model is well trained, the samples from five datasets need to be preprocessed. According to the sample format described in “Problem definition”, we mainly use the values of four fields mentioned above in samples from each dataset. Then, we use a Pandas tool to preprocess the original samples from each dataset and extract four attributes, including userID, itemID, user’s reviews on the item, and user’s rating on the item (1–5 points). As a result, every sample is unified as a userID-itemID-review-rating quadruplet by preprocessing to facilitate the input model for training.

#### Evaluation metrics

We leverage mean square error (MSE) and mean absolute error (MAE) to evaluate the performance of different methods. The two metrics are utilized to measure the accuracy of rating prediction by computing the difference between predicted and actual ratings. Lower MSE and MAE values indicate higher accuracy of model prediction. The formulas for calculating MSE and MAE are:

15 $$\begin{aligned} {\text {MSE}}= & {} \frac{1}{{|T|}}\sum \limits _{a \in T} {{{(\hat{y}(a) - y(a))}^2}} \end{aligned}$$

16 $$\begin{aligned} {\text {MAE}}= & {} \frac{1}{{|T|}}\sum \limits _{a \in T} {|\hat{y}(a) - y(a)|} \end{aligned}$$

where *T* represents the test samples, |*T*| represents the number of samples in the test set, $\hat{y}(a)$ denotes the predicted rating of a test sample *a*, *y*(*a*) is the real rating of sample *a* from the corresponding test dataset.

#### Baseline methods

To demonstrate the effectiveness of our AFMRUI model, we select a traditional recommendation model based on matrix factorization and nine models based on neural networks. The selected representative baseline methods are described as follows.

- **M**atrix **F**actorization (**MF**)^39^: This method is a regression algorithm, which only takes rating data as input, and obtains user and item features by matrix factorization.
- **Deep** **Co**operative **N**eural **N**etworks (**DeepCoNN**)^14^: This model utilizes two parallel convolutional layers to process review documents for users and items, respectively, and uses FM to perform rating prediction, which shows that review information can alleviate the sparsity problem of rating data.
- **D**ual **Att**ention-based **n**etwork (**D-Attn**)^15^: This model obtains review-based feature representations of users and items by combining local and global learning, and finally predicts ratings by using dot product.
- **Trans**formational Neural **Net**work**s** (**TransNets**)^40^: This model adds a transform layer to DeepCoNN, which mainly transforms the latent representations of reviews into user and item features, and uses FM to predict ratings.
- **N**eural **A**ttentional **R**egression Model with **R**eview-level **E**xplanations (**NARRE**)^16^: This model learns user and item features using CNN and attention mechanism, and uses LFM for rating prediction.
- **M**ulti-**P**ointer **C**o-attention **N**etworks (**MPCN**)^28^: This model uses a pointer network to learn user and item features from reviews and uses FM for rating prediction.
- **D**ual **A**ttention **M**utual **L**earning (**DAML**)^17^: This model utilizes local and mutual attention of CNN to jointly learn user and item features from reviews, and neural factorization machine is introduced to predict ratings.
- **N**eural **C**ollaborative **E**mbedding **M**odel (**NCEM**)^41^: This model utilizes an aspect-level attention layer to measure the correlation degree of reviews towards different aspects, and a multi-layer neural factorization machine is introduced to predict ratings.
- **C**ross-domain Recommendation Framework Via **A**spect **T**ransfer **N**etwork (**CATN**)^42^: The model learns the aspect level features of each user and item from the corresponding reviews through attention mechanism, then semantic matching is performed between such aspect level features to predict ratings.
- **M**atch **P**yramid **R**ecommender **S**ystem (**MPRS**)^43^: This model uses a CNN architecture fed by the matching matrix of corresponding reviews for a pair of user–item, and a regression layer is introduced to predict ratings.

#### Configuration

In our experiments, the code was written in Python 3.8, and TensorFlow 1.15.5 was utilized as a framework. All experiments were conducted on a Linux server with Intel(R) Xeon(R) Gold 6330 CPU and RTX 3090 24 GB GPU. We randomly divided each dataset used in the experiments into training set, validation set and test set according to the proportion of 8:1:1. Furthermore, we selected parameters on the validation set and performed evaluation on the test set. The settings of other parameters are described as follows:

- For MF^39^ method, the latent dimensions of users and items are uniformly set to 50.
- For DeepCoNN^14^, D-Attn^15^, TransNets^40^, NARRE^16^, MPCN^28^, DAML^17^, NCEM^41^, CATN^42^ and MPRS^43^ , we set the parameters for the methods based on the setting strategies in the corresponding paper. More specifically, learning rate is 0.002, dropout is set from $\{$0.1, 0.3, 0.5, 0.7, 0.9$\}$, and batch size is set from $\{$32, 64, 128, 256, 512$\}$ to find the best parameters. The ID embedding dimension is set to 32 in NARRE and DAML model; in D-Attn, NARRE, DAML, NCEM and CATN models, the dimension of the attention score vector is set to 100; in DeepCoNN, TransNets, NARRE, CATN and MPRS models, CNN is used to process reviews, where the size of each convolution kernel is set to 3, and the number of convolution kernel is set to 50; the word vector model adopted is Glove and the embedding dimension is 100; in NCEM, the version of BERT is “BERT-base”. Note that if FM is used in any model, the latent dimension is set to 32.
- For our proposed model AFMRUI, we carefully tested batch size from $\{$32, 64, 128, 256, 512$\}$ and looked for the optimal value of learning rate from $\{$0.0001, 0.0005, 0.001, 0.005$\}$ for each dataset. To prevent overfitting, we turned dropout from $\{$0.1, 0.3, 0.5, 0.7, 0.9$\}$. Then, batch size is set to 512, learning rate is set to 0.001, dropout is set to 0.3, and Adam is used as the optimizer. The unified maximum length of reviews is set to 100. The version of RoBERTa is “RoBERTa-base”, where we subsequently add a fully connected layer to compress the semantic feature dimension *c*. The hidden unit number ${t_1}$ is set to 50 in attention layer. The size *d* of embedding vector is set to 6 in rating prediction layer. The other parameters are determined by optimizing MSE and MAE on a validation set from each dataset.

### Results and discussions

#### Comparison of model performance

In this subsection, we compare the performance of eleven methods on five datasets. Table 3 shows the results, with the best-performing ones highlighted in bold. From Table 3, we can make the following observations.

**Table 3:** Performance comparison on five datasets (mean ± std).

	Params (M)	Digital music		Baby		Office products		Beauty		Yelp	
		MSE	MAE	MSE	MAE	MSE	MAE	MSE	MAE	MSE	MAE
MF^39^	0.126	1.956 ± 0.002	1.204 ± 0.009	1.755 ± 0.001	1.320 ± 0.005	1.143 ± 0.008	0.996 ± 0.009	1.950 ± 0.004	1.381 ± 0.006	1.828 ± 0.009	1.526 ± 0.005
DeepCoNN^14^	6.303	1.202 ± 0.009	0.722 ± 0.046	1.440 ± 0.005	0.873 ± 0.037	0.909 ± 0.003	0.707 ± 0.004	1.453 ± 0.015	0.922 ± 0.008	1.687 ± 0.003	1.361 ± 0.028
D_Attn^15^	9.152	1.014 ± 0.015	0.697 ± 0.007	1.325 ± 0.004	0.849 ± 0.001	0.815 ± 0.006	0.754 ± 0.005	1.419 ± 0.009	0.845 ± 0.007	1.651 ± 0.003	1.358 ± 0.009
TransNets^40^	15.373	1.055 ± 0.004	0.701 ± 0.002	1.334 ± 0.005	0.853 ± 0.003	0.824 ± 0.005	0.746 ± 0.005	1.412 ± 0.005	0.841 ± 0.007	1.623 ± 0.005	1.119 ± 0.003
NARRE^16^	11.297	0.965 ± 0.002	0.686 ± 0.005	1.312 ± 0.009	0.851 ± 0.006	0.817 ± 0.021	0.727 ± 0.004	1.396 ± 0.007	0.828 ± 0.002	1.571 ± 0.006	1.014 ± 0.007
MPCN^28^	11.879	0.970 ± 0.005	0.729 ± 0.004	1.304 ± 0.007	0.858 ± 0.005	0.779 ± 0.004	0.670 ± 0.004	1.386 ± 0.008	0.894 ± 0.001	1.608 ± 0.017	1.106 ± 0.007
DAML^17^	14.004	0.959 ± 0.021	0.705 ± 0.003	1.298 ± 0.002	0.853 ± 0.005	0.791 ± 0.007	0.689 ± 0.013	1.379 ± 0.007	0.843 ± 0.004	1.581 ± 0.009	1.052 ± 0.008
NCEM^41^	110.334	0.956 ± 0.008	0.691 ± 0.012	1.290 ± 0.018	0.851 ± 0.002	0.788 ± 0.003	0.667 ± 0.002	1.370 ± 0.002	0.816 ± 0.001	1.567 ± 0.002	1.001 ± 0.001
CATN^42^	32.193	0.952 ± 0.013	0.678 ± 0.002	1.285 ± 0.005	0.847 ± 0.007	0.774 ± 0.002	0.655 ± 0.011	1.366 ± 0.003	0.806 ± 0.003	1.554 ± 0.001	0.993 ± 0.001
MPRS^43^	18.137	0.947 ± 0.002	0.678 ± 0.006	1.282 ± 0.005	0.845 ± 0.002	0.772 ± 0.010	0.653 ± 0.009	1.361 ± 0.005	0.800 ± 0.006	1.548 ± 0.004	0.981 ± 0.015
AFMRUI	**127.452**	**0.910** ± **0.002**	**0.657** ± **0.009**	**1.256** ± **0.003**	**0.821** ± **0.008**	**0.740** ± **0.007**	**0.638** ± **0.001**	**1.341** ± **0.010**	**0.786** ± **0.004**	**1.502** ± **0.003**	**0.954** ± **0.009**

First, our proposed model, AFMRUI, outperforms other models in terms of MSE and MAE on five datasets. Notably, when compared with the best baseline method (MPRS), AFMRUI enhances performance on Digital Music dataset by approximately 3.7$\%$ for MSE and 2.1$\%$ for MAE. Similarly, high performance gains are observed on the other four datasets. These results demonstrate the superiority of our model.

Second, methods utilizing review information generally work better than those that only consider the rating data. It is clear that, DeepCoNN, D-Attn, TransNets, NARRE, MPCN, DAML, NCEM, CATN, MPRS and AFMRUI perform better than MF in terms of MSE and MAE on five datasets. The performance improvements of these methods may be due to leveraging neural networks for rating prediction by using review information, which can effectively capture user/item features from review information, and reduce the effect of data sparsity due to only using rating data. Therefore, these methods utilizing review information gain pure improvement compared with MF.

Third, our proposed AFMRUI model performs better than nine baseline models leveraging review information on five datasets. The reason is that, in our model, RoBERTa can capture global context and mitigate the problem of polysemy in user/item reviews, in which the accurately understanding of review information is guaranteed. Moreover, our model uses AFM, rather than dot product and FM, to learn different feature interactions and further to distinguish the importance of different feature interactions, which can also alleviate the effect of noise that may be introduced by useless feature interactions, so that AFMRUI achieves better performance on five datasets.

In addition, for each of these eleven methods, we also provide an order of magnitude of approximate model parameters for comparison, as shown in the second column in Table 3, where M represents millions. The comparison results from Table 3 show that ten deep learning-based methods have more parameters compared with MF, mainly due to the fact that deep learning models usually contain a multi-layer neural network, and each layer contains a large number of parameters. While NCEM and AFMRUI have much more model parameters compared with the other eight deep learning-based methods, mainly because both methods use pre-trained models to encode reviews, and pre-trained models need to learn a lot of linguistic knowledge and laws to have stronger expression and generalization ability. Compared with NCEM, AFMRUI has more model parameters, mainly because our model leverages the pre-trained model RoBERTa, which has been made improvements in model structure and optimization algorithms on the basis of BERT used in NCEM, thus requiring more parameters than NCEM.

#### Effectiveness of different components

In this subsection, we performed ablation experiments to analyze the effects of different components to model performance.

In order to validate the benefits brought by each component, we construct the following variants of AFMRUI based on the basic model, AFMRUI-base, which uses static word vector model Glove to represent user/item review embedding features and predicts user’s rating on an item by FM.

- AFMRUI-Ro: This model uses RoBERTa instead of Glove to obtain user/item review embedding features on the basis of AFMRUI-base. This variant model is to verify that RoBERTa is better than Glove in extracting review embedding features.
- AFMRUI-Bi: In this model, BiGRU is added on the basis of AFMRUI-Ro to encode each user/item review embedding features output from RoBERTa. This variant model is to verify the effectiveness of BiGRU.
- AFMRUI-Att: This model adds an attention network on the basis of Review-Bi, and this variant model is to verify the effectiveness of the attention network in measuring the contribution of each review to user/item feature representation.

**Table 4:** Comparison of models with different components.

Models	RoBERTa	BiGRU	Attention	AFM
AFMRUI-base	$\backslash$	$\backslash$	$\backslash$	$\backslash$
AFMRUI-Ro	$\checkmark$	$\backslash$	$\backslash$	$\backslash$
AFMRUI-Bi	$\checkmark$	$\checkmark$	$\backslash$	$\backslash$
AFMRUI-Att	$\checkmark$	$\checkmark$	$\checkmark$	$\backslash$
AFMRUI	$\checkmark$	$\checkmark$	$\checkmark$	$\checkmark$

**Table 5:** Effectiveness of different components on five datasets.

	Digital music		Baby		Office products		Beauty		Yelp	
	MSE	MAE	MSE	MAE	MSE	MAE	MSE	MAE	MSE	MAE
AFMRUI-base	1.025	0.722	1.331	0.880	0.844	0.695	1.417	0.882	1.714	1.353
AFMRUI-Ro	0.968	0.681	1.309	0.856	0.796	0.672	1.392	0.822	1.605	1.121
AFMRUI-Bi	0.957	0.667	1.291	0.844	0.783	0.661	1.377	0.816	1.564	1.047
AFMRUI-Att	0.943	0.675	1.274	0.830	0.766	0.650	1.365	0.806	1.537	0.991
AFMRUI	**0.910**	**0.657**	**1.256**	**0.821**	**0.740**	**0.638**	**1.341**	**0.786**	**1.502**	**0.954**

Significant values are in [bold].

Table 4 shows the models with different components. We take two metrics to demonstrate the effectiveness of the models from Table 4 on five datasets. The results are shown in Table 5.

It can be seen from Table 5, the model performance of AFMRUI-Ro has been improved compared with the basic model, indicating that using RoBERTa to obtain context-related user/item review embedding features, which can alleviate the problem of polysemy and effectively enhance the feature representation. Compared with AFMRUI-Ro, AFMRUI-Bi performs better mainly because BiGRU is more suitable for dealing with sequence problems and can fully exploit the internal dependencies among reviews. While the performance of AFMRUI-Bi is worse than AFMRUI-Att, because the attention network introduced can adaptively measure the importance of each review to user/item feature representation, enabling the model to focus on more useful reviews.

In contrast, the performance of our proposed AFMRUI model is better than the other four variant models, which shows that AFM can better learn the feature interactions of users and items to obtain more accurate prediction rating, and also demonstrates that integrating these components can help to better model review features of users and items, so as to improve the model performance.

### Effect of parameters

In this section, we analyzed the effects of different model parameters on the performance of AFMRUI. Here, we focused on five critical parameters, namely, the maximum number of user reviews *n* and item reviews *m*, the semantic feature dimension *c*, GRU hidden dimension *l* and the latent dimension *k*. Next, we analyzed the effects of five parameters on two metrics.

#### Effect of maximum number of reviews

The proposed AFMRUI model performs rating prediction based on user reviews and item reviews. Therefore, the maximum number of user reviews *n* and item reviews *m* directly affects the feature representations of users and items, thereby affecting the model performance. Considering that different datasets have different numbers of reviews for different users and different items, so we make statistics on the number of user reviews and item reviews from five datasets to determine the range for the maximum number of reviews, as shown in Table 6.

**Table 6:** Statistics of reviews from five datasets.

Datasets	Number of users	Percentage of total users (%)	Number of items	Percentage of total items (%)
Digital music	4449 ($n\le$ 13)	80.29	2892 ($m\le$ 20)	81.05
Baby	15,991 ($n\le$ 10)	82.23	5666 ($m\le$ 28)	80.37
Office products	4024 ($n\le$ 10)	82.04	1979 ($m\le$ 11)	81.78
Beauty	18,117 ($n\le$ 10)	81.01	9712 ($m\le$ 19)	80.26
Yelp	932,169 ($n\le$ 15)	81.48	139,767 ($m\le$ 20)	80.32

**Figure 3:**
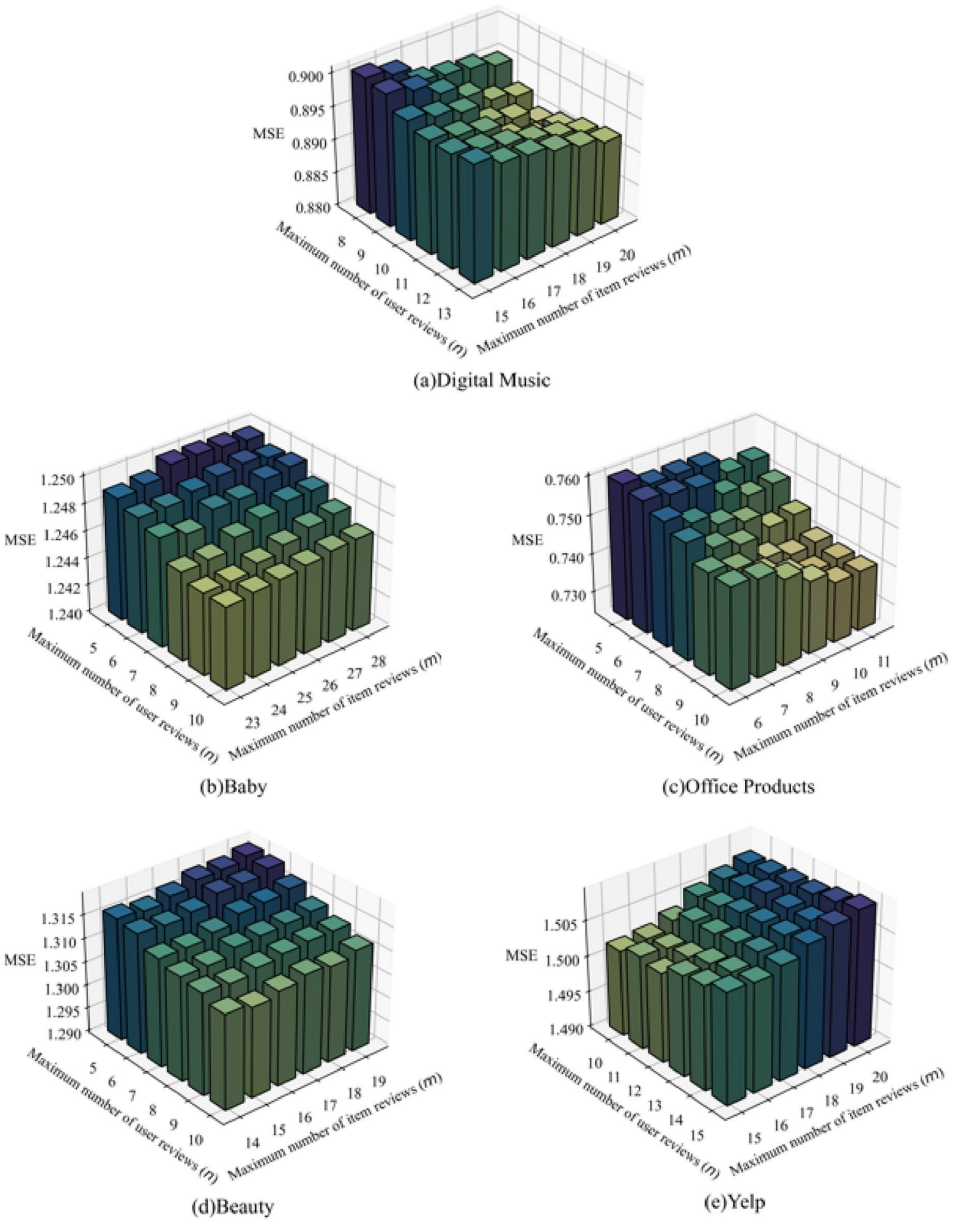
Effect of maximum number of user reviews and item reviews on model performance.

Take digital music dataset (the second row in Table 6) as an example, 4449 users have up to 13 reviews, accounting for 80.29$\%$ of the total number of users, and 2892 items have up to 20 reviews, accounting for 81.05$\%$ of the total number of items. According to the statistical results, considering that the noise will be introduced if the number of reviews is too large, and less effective information is extracted if the number of reviews is too small, so we set the range for maximum number of user reviews to $\{$8, 9, 10, 11, 12, 13$\}$, and the range for maximum number of item reviews to $\{$15, 16, 17, 18, 19, 20$\}$. Similarly, we set the ranges for maximum number of reviews from the other four datasets while keeping other hyper-parameters unchanged. Figure 3 shows the results on five datasets. Since the results on MAE are similar to that on MSE, so we take MSE as an example to analyze the effects of the parameters on model performance.

As shown in Fig. 3a, for digital music dataset, with the increase of *n* and *m*, MSE decreases first and then increases. This is because when the number of reviews is too large, noise may be introduced to affect the feature representations of users and items. While the number of reviews is too small to accurately express the feature representations of users and items. Therefore, we set the maximum number of user reviews *n* to 10 and set the maximum number of item reviews *m* to 20 that can get the best performance on digital music dataset. Similarly, the maximum number of user reviews and item reviews are set to *n* = 10, m = 23 on Baby dataset, respectively; for office products dataset, *n* = 8 and *m* = 10; for beauty dataset, *n* = 10 and *m* = 15; for Yelp, *n* = 10 and *m* = 15. According to the above analysis, we select such values as the corresponding maximum numbers of user reviews and item reviews on five datasets.

#### Effect of semantic feature dimension *c*

**Figure 4:**
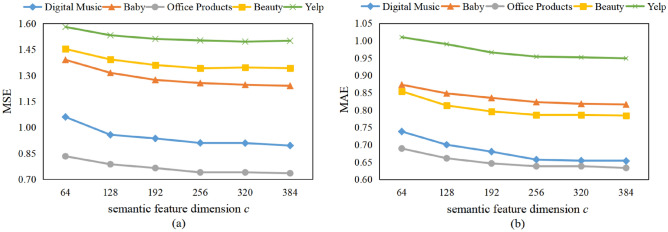
Effect of semantic feature dimension *c* on model performance.

In order to investigate how sensitive AFMRUI is to the semantic feature dimension *c*, we fixed the dimension of the review embedding feature output by RoBERTa to 768, and further obtained the corresponding review embedding features with different semantic feature dimension *c* through fully connected layer compression. We demonstrated the effects of *c* on five datasets in Fig. 4. As shown in Fig. 4, for five datasets, with the increase of *c*, the model performance is gradually improved. When *c* is 256, the model performance reaches the best, and then begins to decline. Moreover, the computational cost is also increasing. Therefore, we set the semantic feature dimension *c* to 256 that can get the best performance on five datasets.

**Figure 5:**
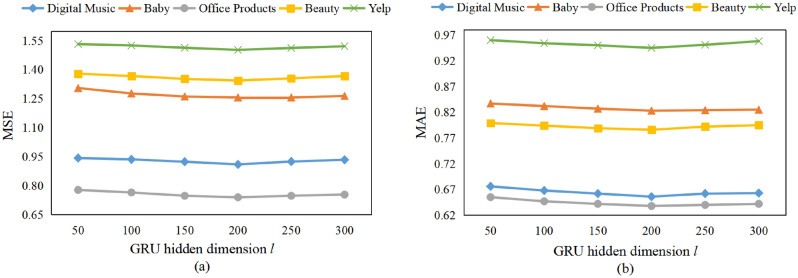
Effect of GRU hidden dimension *l* on model performance.

**Figure 6:**
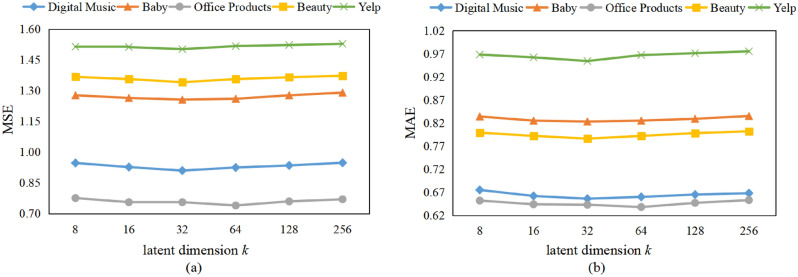
Effect of latent dimension *k* on model performance.

#### Effect of GRU hidden dimension *l*

To illustrate the effect of GRU hidden dimension *l*, we set values of *l* as 50, 100, 150, 200, 250, 300 while keeping other hyper-parameters unchanged. Figure 5 shows the results on five datasets. The curves show the trend of falling first and then rising on five datasets. This maybe because when GRU hidden dimension is too small, it cannot fully mine the internal dependencies among review embedding features. While when GRU hidden dimension is too large, it will make the model over-fitting. Therefore, similar to selection of the semantic dimension *c*, we set GRU hidden dimension to 200 that can get the best performance on five datasets.

#### Effect of latent dimension *k*

In this subsection, we investigate the impact of latent dimension *k* on model performance while keeping other parameters unchanged. The results are presented in Fig. 6. We observe that as *k* increases, MSE and MAE first decrease for digital music, baby, beauty and Yelp datasets, reach the best when *k* is 32, and increase thereafter. For office products dataset, MSE and MAE reach the best when *k* is 64. This is because a small value of *k* may lead to the model being unable to capture all potential information from user and item reviews, while a large value of *k* may cause over-fitting and increase the model complexity. Therefore, we set *k* to 64 on Office Products dataset and 32 on the other four datasets.

### Comparison of different embedding representation methods

In this section, we discuss the impact of different embedding representation methods on the model performance. Here, we select a classical algorithm DeepCoNN^14^ and the best baseline method MPRS^43^ with different embedding representations. As shown in Table 7, we mainly discuss nine comparison methods.

**Table 7:** Effect of different embedding representation methods on model performance.

	Digital music		Baby		Office products		Beauty		Yelp	
	MSE	MAE	MSE	MAE	MSE	MAE	MSE	MAE	MSE	MAE
DeepCoNN-Glove^14^	1.202	0.722	1.440	0.873	0.909	0.707	1.453	0.922	1.687	1.361
DeepCoNN-BERT-base	1.185	0.706	1.417	0.869	0.873	0.684	1.424	0.907	1.650	1.322
DeepCoNN-RoBERTa-base	1.172	0.698	1.403	0.856	0.856	0.675	1.406	0.897	1.633	1.309
MPRS-Glove^43^	0.947	0.678	1.282	0.845	0.772	0.653	1.361	0.800	1.548	0.981
MPRS-BERT-base	0.925	0.676	1.262	0.833	0.760	0.648	1.358	0.804	1.537	0.971
MPRS-RoBERTa-base	0.919	0.664	1.258	0.827	0.756	0.644	1.350	0.798	1.525	0.964
AFMRUI-Glove	0.934	0.675	1.280	0.845	0.767	0.658	1.365	0.809	1.540	0.979
AFMRUI-BERT-base	0.918	0.662	1.266	0.827	0.751	0.644	1.347	0.799	1.517	0.965
AFMRUI	**0.910**	**0.657**	**1.256**	**0.821**	**0.740**	**0.638**	**1.341**	**0.786**	**1.502**	**0.954**

Significant values are in [bold].

The experimental results reported in Table 7 shows that our proposed model, AFMRUI, outperforms its variants, AFMRUI-Glove and AFMRUI-BERT-base, in terms of MSE and MAE on all five datasets. Specifically, on the Yelp dataset, AFMRUI improves performance by approximately 3.8% on MSE and 3.5% on MAE compared with AFMRUI-Glove; and the relative performance improvements are 1.5% on MSE and 1.1% on MAE compared with AFMRUI-BERT-base. The other four datasets show similarly high performance gains. These results essentially demonstrate the competitiveness of the proposed model using RoBERTa to obtain context-related user/item review embedding features, which can alleviate the problem of polysemy and effectively enhance the feature representation.

In addition, we also compared DeepCoNN^14^, MPRS^43^, and their variant models. The experimental results show that DeepCoNN-BERT-base and DeepCoNN-RoBERTa-base outperform DeepCoNN-Glove, MPRS-BERT-base and MPRS-RoBERTa-base outperform MPRS-Glove, mainly because the traditional word vector model cannot rely on the before-and-after review information in the review set for efficient representations of users and items. However, BERT-base and RoBERTa-base can alleviate this problem. Whereas DeepCoNN-RoBERTa-base outperforms DeepCoNN-BERT-base, MPRS-RoBERTa-base outperforms MPRS-BERT-base, mainly because RoBERTa-base not only inherits the advantages of BERT-base, but also uses new hyperparameters and a new large dataset for retraining. Not only does it alleviate the problem of multiple meanings of words in reviews, but it also better models the global information and semantic representations of user and item reviews, resulting in more accurate predictive scores and better model performance.

### Comparison of different feature interaction methods

In this section, we discuss the impact of different feature interaction methods on the model performance. We mainly discuss the following three methods.

**Table 8:** Effect of different feature interaction methods on model performance.

	Digital music		Baby		Office products		Beauty		Yelp	
	MSE	MAE	MSE	MAE	MSE	MAE	MSE	MAE	MSE	MAE
AFMRUI-dp	0.968	0.681	1.301	0.844	0.785	0.661	1.380	0.816	1.564	1.019
AFMRUI-FM	0.943	0.675	1.274	0.830	0.766	0.650	1.365	0.806	1.535	0.991
AFMRUI	**0.910**	**0.657**	**1.256**	**0.821**	**0.740**	**0.638**	**1.341**	**0.786**	**1.502**	**0.954**

Significant values are in [bold].

- AFMRUI-dp: The method conducts dot product operation on user review embedding and item review embedding to predict rating.
- AFMRUI-FM: This approach encodes a vector formed by concatenating user and item review embeddings through FM.
- AFMRUI: Our proposed method, uses AFM to learn the feature interactions of users and items to perform rating prediction.

Table 8 shows the results on five datasets. It can be seen from Table 8, AFMRUI-dp experiences the most performance decrease compared with AFMRUI-FM and AFMRUI on five datasets, whereas AFMRUI has the best performance. This is because dot product operation used by AFMRUI-dp cannot fully explore the complex internal structure of the joint vector of user review embedding and item review embedding. While the advantage of FM over dot product operation is that it can capture feature interactions between any two dimensions in the joint vector of user review embedding and item review embedding. Thus, the performance of AFMRUI-FM is better than AFMRUI-dp.

Compared with AFMRUI-FM, our AFMRUI model is more effective because AFM in our model adds attention mechanism on the basis of FM, and it can further distinguish the importance of different feature interactions, which can alleviate the effect of noise possibly introduced by useless feature interactions, so as to obtain more accurate prediction rating and then improve the model performance.

**Table 9:** User–item feature interaction type.

Types of feature interactions	Interaction object
*U*–*U*	User–user
*U*–*I*	User–item
*I*–*I*	Item–item

**Figure 7:**
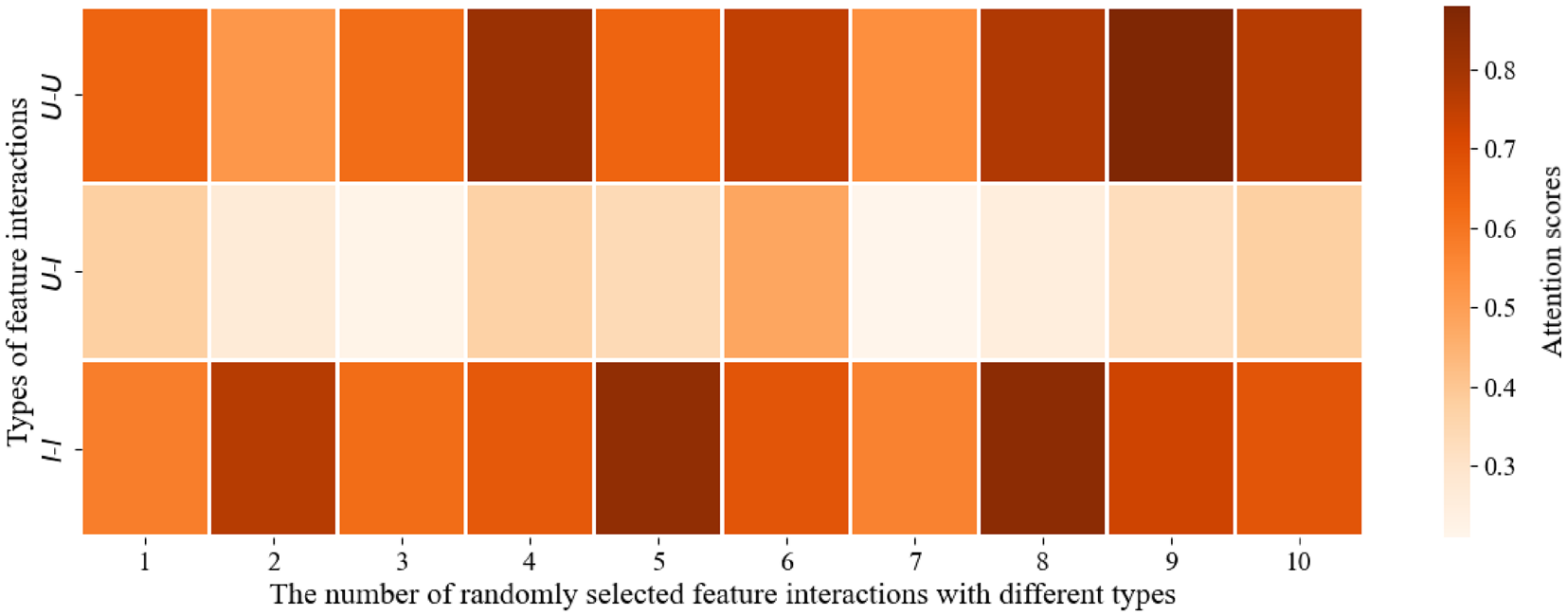
Attention scores of feature interactions with different types.

On the basis of above analysis, in order to further explore the contribution of different feature interactions in our AFMRUI model more intuitively, we use Digital Music dataset as an example to demonstrate the contributions of different feature interactions. Since our AFMRUI model achieves the best results on the Digital Music dataset when the number of latent dimensions *k* is 32, the dimensions of both user review embedding ${\text {R}_u}$ and item review embedding ${\text {R}_i}$ is set to 32, and the dimension of vector x stitched together from them is 64, i.e., $\mathrm{{x}} =({\mathrm{{R}}_u},{\mathrm{{R}}_i}) =$ (${x_{1}}$-${x_{32}}$, ${x_{33}}-{x_{64}}$). Where ${x_1}-{x_{32}}$ is defined as user interaction object *U* and ${x_{33}}-{x_{64}}$ is defined as item interaction object *I*, so there are three types of feature interactions in vector x, as shown in Table 9. A user–item feature interaction (e.g., $x_1$ $x_{33}$) can be formed by taking a random dimension from *U* and *I*. Repeatedly, we select 10 different user–item feature interactions with feature interaction type *U*–*I*. Similarly, we obtain 10 different feature interactions with the other two types, respectively. The attention scores of these feature interactions are shown in Fig. 7.

As shown in Fig. 7, the lighter the color, the lower the attention score and the less it contributes to the model performance, and vice versa. Specifically, the feature interaction type *U*–*I*, which has been adopted by models such as DeepCoNN^14^ and TransNets^40^, achieved good results, indicating that user–item feature interactions are beneficial for the quality of rating prediction. However, according to Fig. 7, it can be seen that the attention scores for *U*–*I* feature interactions are stable between 0.2 and 0.5, indicating that not all user–item feature interactions have positive impacts on the rating prediction. While the other types of *U*–*U* and *I*–*I*have more higher attention scores, mainly in the range of 0.5–0.9, indicating that although they are the same interaction objects, the feature interactions between them are more important and can have positive impacts on the model performance, resulting in more accurate prediction of user’s rating of an item, and thus provide better recommendation.

In summary, it can be seen that different feature interactions have different attention scores and have different impacts on model performance. While AFM adopted in our model can distinguish the importance of different feature interactions through the obtained attention scores, thereby alleviating the impact of useless feature interactions on model performance.

## Conclusions

In recent years, the review-based recommendation is one of hot research topics in recommender systems. In this paper, we proposed an AFMRUI model for recommendation. Specifically, AFMRUI leverages RoBERTa to mitigate the problem of polysemy in user/item reviews, and learns reviews embedding of users and items through BiGRU and attention network, so as to better model user review embedding and item review embedding. Then it utilizes AFM to learn user–item feature interactions, which can obtain more accurate prediction rating by distinguishing the importance of different feature interactions. Extensive experiments on five publicly available datasets have demonstrated that the proposed AFMRUI model outperforms the state-of the-art methods regarding two metrics.

In this paper, we just leverage review information to extract users and items features. Recently, studies have shown that user–item interaction graph^44,45^ has additional useful information to promote recommendation. Therefore, in the future work, we will combine review information with user–item interaction graph to capture more accurate features of users and items, so as to provide better model performance.

## Data Availability

The data used to support the findings of this study are available from http://jmcauley.ucsd.edu/data/amazon/ and https://www.yelp.com/dataset.

## Code availability

The source code of the proposed model is publicly available for download at Github: https://github.com/Jindidi/AFMRUI.git.
